# MBNL1-dependent alternative splicing promotes neuronal differentiation through regulation of NUMA1 exon 16 during fibroblast-to-neuron reprogramming

**DOI:** 10.3389/fcell.2026.1862147

**Published:** 2026-06-22

**Authors:** Jun Li, Qiu-Shuang Long, Ruo-Qi Zhang, Bing-Lin Zhu

**Affiliations:** 1 Brain Research Center and State Key Laboratory of Trauma, Burns, and Combined Injury, The Army Medical University (Third Military Medical University), Chongqing, China; 2 Institute of Advanced Pathology Research, Jinfeng Laboratory, Chongqing, China

**Keywords:** alternative splicing, MBNL1, neuronal reprogramming, NUMA1, post-transcriptional regulation

## Abstract

**Introduction:**

Direct neuronal reprogramming enables the generation of neurons from somatic cells without passing through a pluripotent state, yet the post-transcriptional mechanisms that refine neuronal identity after fate induction remain poorly understood.

**Methods:**

We examined alternative splicing during fibroblast-to-neuron reprogramming and investigated the effects of MBNL1 knockdown on neuronal phenotype, transcriptomic and splicing changes, and NUMA1 exon 16 regulation.

**Results:**

MBNL1 knockdown establishes a distinct reprogramming state (AMmnp) characterized by enhanced neurite outgrowth and a more neuron-like differentiated phenotype, without significantly affecting conversion efficiency. Among MBNL1-dependent transcriptomic and splicing changes, NUMA1 exon 16 emerges as a key target, with exon inclusion reducing neuronal marker expression specifically in the AMmnp context, whereas exon skipping is associated with a more permissive neuronal phenotypic output.

**Discussion:**

Together, these findings position alternative splicing as an active regulatory layer that shapes neuronal identity and phenotypic output during reprogramming, linking MBNL1-dependent splicing control to cytoskeletal remodeling and neuronal differentiation.

## Introduction

Direct conversion of somatic cells into neurons has emerged as a powerful approach for generating neuronal cells and for dissecting molecular principles of cell fate specification (Vasan et al., 2021; Bocchi et al., 2022). Forced expression of neuronal transcription factors, often combined with microRNAs and small-molecule modulation, can efficiently induce neuronal identity from fibroblasts (Cates et al., 2021; Wang et al., 2021; Bocchi et al., 2022). However, neuronal reprogramming yields heterogeneous induced neurons that differ in maturation state, subtype identity, and functional properties (Bocchi et al., 2022; Pereira et al., 2024). How neuronal identity and maturation are refined downstream of transcription factor-driven fate induction remains incompletely understood (Wang et al., 2021; Bocchi et al., 2022).

Post-transcriptional regulation, particularly alternative splicing, plays a central role in neuronal development and functional diversification (Fisher and Feng, 2022; Lee et al., 2023). Dynamic changes in exon usage shape neuronal differentiation, synaptic organization, and circuit formation (Feng et al., 2021; Lee et al., 2023; Liu et al., 2023). These processes are coordinated by RNA-binding proteins, including members of the PTBP, RBFOX, NOVA, and MBNL families, which orchestrate neuron-specific splicing programs during development (Fisher and Feng, 2022; Lee et al., 2023). Consistent with their central regulatory roles, dysregulation of neuronal splicing factors has been implicated in a wide range of neurodevelopmental and neurodegenerative disorders (Nikom and Zheng, 2023; Engal et al., 2024; Li and Sun, 2025).

The Muscleblind-like (MBNL) family of RNA-binding proteins has been extensively studied for its roles in developmentally regulated splicing and in the pathogenesis of myotonic dystrophy (Degener et al., 2022; Zhou et al., 2025). Beyond muscle, MBNL1 is expressed in the nervous system and has been linked to neuronal maturation and synaptic function (Wang et al., 2022; Tahraoui-Bories et al., 2023; Zhou et al., 2025). Previous studies have shown that MBNL1 controls broad alternative splicing networks associated with cellular differentiation, suggesting that splicing regulators can influence cell identity beyond canonical lineage boundaries (Han et al., 2013). Notably, NUMA1 has been identified as a direct splicing target of MBNL1, with *MBNL1* depletion promoting skipping of exon 16 (Sebestyen et al., 2016; Louis et al., 2024). However, whether MBNL1-NUMA1 splicing regulation contributes to neuronal fate acquisition during reprogramming has not been addressed (Zhou et al., 2025).

In this study, we examined the role of MBNL1-dependent alternative splicing during direct fibroblast-to-neuron reprogramming. Compared with our previously established neuronal induction paradigm, AMnp (ASCL1 + miR-9/9*-124 + *PTBP2* and *p53* knockdown; (Zhu et al., 2023)), the *MBNL1*-deficient condition, AMmnp (AMnp with additional *MBNL1* knockdown) exhibited widespread transcriptional and splicing alterations together with enhanced neurite outgrowth. We identify *NUMA1* exon 16 as a key MBNL1-dependent splicing event and show evidence that its inclusion or exclusion modulates neuronal marker output in a context-dependent manner. Together, our findings reveal a functional link between alternative splicing regulation and neuronal identity refinement during reprogramming, highlighting post-transcriptional control as a key determinant of induced neuronal phenotypic states.

## Materials and methods

### Materials

The following plasmids were sourced from Addgene: pLKO.1/p53 shRNA (cat. No. 19119), pMD2. G (cat. No. 12259), psPAX2 (cat. No. 12260), and pTight-9–124-BclxL encoding the miR-9/9*-124 microRNA cassette (cat. No. 60857). Lentiviral constructs targeting PTBP2 (pLKO.1 backbone), as well as FUW-ASCL1 and FUW-M2rtTA, were generated and previously characterized in our laboratory (Jiang et al., 2015; Zhu et al., 2023). The pLKO.1-based shRNA targeting MBNL1 (TRCN0000063964) was obtained from Itskovich et al. (Itskovich et al., 2020). All plasmid constructs were sequence-confirmed by Sanger sequencing prior to use, and plasmid DNA was prepared using the EndoFree Plasmid Maxi Kit (Vazyme, cat. No. DC202-01) for subsequent transfection and lentivirus production.

### Cell culture

Primary human fibroblast lines, including skin fibroblasts HFF-1 (newborn, cat. No. IM-H471) and HSF (adult, cat. No. IM-H032), were acquired from Immocell (Xiamen, China) and were authenticated by short tandem repeat (STR) profiling. STR analysis confirmed a complete match to the HFF-1 and HSF reference profile in the International Cell Line Authentication Committee (ICLAC), with no evidence of cross-contamination.

All fibroblasts were maintained in DMEM (Gibco, cat. No. 11965–092) supplemented with 10% fetal bovine serum (FBS; Gibco, cat. No. 10437028), 0.1 mM NEAA (Gibco, cat. No. 11140050), 2 mM L-glutamine (Gibco, cat. No. 25030081), 100 U/mL penicillin, and 100 μg/mL streptomycin (Gibco, cat. No. 15140122). Fibroblasts were cultured at 37 °C in a humidified atmosphere with 5% CO_2_, and were passaged every 2–3 days and used at passages 5–10 for transdifferentiation experiments. All cell cultures were regularly tested for the absence of *mycoplasma* by PCR.

### Construction of NUMA1 and NUMA1-ΔE16 lentiviral expression vectors

The pDONR223-NUMA1 plasmid containing the full-length human NUMA1 open reading frame (ORF) was obtained from YouBio (cat. No. G119575) and subcloned into the FUW-tetO-LoxP lentiviral expression vector to generate FUW-NUMA1. A NUMA1 exon 16-deleted variant (NUMA1-ΔE16) was generated by site-directed mutagenesis using the Phusion™ Site-Directed Mutagenesis Kit (Thermo Fisher Scientific, cat. No. F541) according to the manufacturer’s instructions. High-purity 5′-phosphorylated primers flanking exon 16 were used to introduce precise deletion of exon 16 from the pDONR223-NUMA1 template. PCR amplification was performed using Phusion Hot Start II High-Fidelity PCR Master Mix (Thermo Fisher Scientific, cat. No. F565 L), followed by DpnI digestion (New England Biolabs, cat. No. R0176 V) to remove the parental methylated plasmid. The resulting product was verified by agarose gel electrophoresis and subcloned into the FUW-tetO-LoxP vector to generate FUW-NUMA1-ΔE16. All constructs were verified by Sanger sequencing before lentiviral packaging. Primer sequences used for site-directed mutagenesis are listed in Supplementary Table S1.

### Lentivirus packaging and production

Lentiviral particles were produced using a standard packaging protocol with minor modifications, as previously described (Zhu et al., 2023). HEK293FT cells were plated at a density of 5 × 10^6^ cells per 10-cm dish and cultured overnight to reach approximately 90% confluency. Cells were co-transfected together with the indicated transfer plasmid (pLKO.1- or FUW-based constructs) using Lipofectamine 2000 (Thermo Fisher Scientific, cat. No. 11668019) according to the manufacturer’s instructions. Sixteen hours after transfection, the medium was replaced to remove residual transfection reagents. Viral supernatants were collected at 24, 40, and 48 h post-transfection, pooled, clarified by centrifugation at 300 *g* for 5 min, and filtered through a 0.45 μm PVDF syringe filter (Merck, cat. No. SLHVR33RS). Viral titers were determined using an HIV-1 p24 ELISA kit (Abcam, cat. No. ab218268). Lentiviral aliquots were stored at −80 °C and used either fresh or after a single freeze-thaw cycle for subsequent transdifferentiation experiments.

### Transdifferentiation of human fibroblasts

Human fibroblast cell lines HFF-1 and HSF were seeded onto Matrigel-coated 12-well plates (Corning, cat. No. 354277) at a density of 5 × 10^4^ cells per well and cultured overnight at 37 °C in a humidified incubator with 5% CO_2_. The next day, cells were transduced with the indicated lentiviral constructs at a multiplicity of infection (MOI) of 10 for each virus in the presence of 8 μg/mL polybrene (Sigma, cat. No. H9268) to facilitate viral entry. After 16 h, viral supernatants were removed and cultures were washed three times with serum-free DMEM/F12 to eliminate residual virus and serum components. Cells were then maintained in serum-free DMEM/F12 for an additional 24 h to promote G1/S-phase synchronization, a condition that enhances cellular responsiveness to neuronal reprogramming cues (Zhu et al., 2023).

Neuronal induction was initiated the following day (Day 0) by replacing the medium with a defined neural induction medium consisting of DMEM/F12 supplemented with 1 × N2 (Gibco, cat. No. 17502048), 1 × B27 (Gibco, cat. No. 12587010), 0.1 mM non-essential amino acids, 0.5 μM dorsomorphin (Selleck, cat. No. S7840), 2.5 μM SB431542 (Selleck, cat. No. S1067), 1 μM PD0332991 (Selleck, cat. No. S1579), 3 μM CHIR99021 (Selleck, cat. No. S1263), 0.2 mM vitamin C (Sigma, cat. No. A4544), 10 μM Y-27632 (Selleck, cat. No. S6390), 0.5 mM dibutyryl-cAMP (Selleck, cat. No. S7858), and 20 ng/mL each of NGF, GDNF, and BDNF (PeproTech, cat. Nos. 450–01, 450–10, and 450–02), together with 1 μg/mL doxycycline (Selleck, cat. No. S5159) to induce transgene expression. Cultures were maintained under hypoxic conditions (5% O_2_, 5% CO_2_) at 37 °C, and the induction medium was refreshed every other day throughout the reprogramming period.

### Immunofluorescence staining

Cells were fixed in 4% paraformaldehyde (PFA; Sigma, cat. No. 158127) for 20 min at room temperature and subsequently permeabilized with 0.3% Triton X-100 (Sigma, cat. No. T8787) for 20 min. To reduce non-specific antibody binding, samples were blocked with 5% bovine serum albumin (BSA) (Sigma, cat. No. A7030) in phosphate-buffered saline (PBS) for 1 h at room temperature. Primary antibodies diluted in blocking buffer were applied and incubated overnight at 4 °C. The following primary antibodies were used: anti-MAP2 (Santa Cruz, cat. No. sc74421, 1:1,000), anti-TUJ1 (Abcam, cat. No. ab78078, 1:2000), anti-NeuN (Proteintech, cat. No. 26975-1-AP, 1:400), anti-GABA (Sigma, cat. No. A2052 and cat. No. A0310, 1:1,000), anti-TH (ThermoFisher Scientific, cat. No. OPA1-04050, 1:1,000), anti-vGLUT1 (Proteintech, cat. No. 55491-1-AP, 1:500), anti-5HT (Sigma, cat. No. S5545, 1:1,000), anti-DARPP32 (Abcam, cat. No. ab40801, 1:1,000), anti-Parvalbumin (ABclonal, cat. No. A2791, 1:500), anti-SST (ABclonal, cat. No. A9274, 1:500), and anti-5HT3aR (ABclonal, cat. No. A5647, 1:500). After thorough washing with PBS, cells were incubated for 1 h at room temperature with the appropriate Alexa Fluor-conjugated secondary antibodies (ThermoFisher Scientific, cat. Nos. A21121 and A11035, 1:1,000). Nuclear staining was performed using DAPI (ThermoFisher Scientific, cat. No. D1306) for 5 min.

Fluorescence images were acquired using an Olympus IX73 fluorescence microscope, with identical exposure settings applied across all experimental conditions. For quantitative analysis, 15 randomly selected fields per condition (n = 15) were analyzed using ImageJ (NIH). These fields were derived from three independent cell preparations, each representing a separate round of plating, viral transduction, and reprogramming.

Marker-positive cells were defined based on fluorescence intensity exceeding background levels, with uniform threshold settings applied to all images. Cell counts were performed using the ImageJ Cell Counter plugin, and results were expressed either as absolute numbers or as a percentage of DAPI^+^ nuclei. Neurite length was quantified in ImageJ using the Simple Neurite Tracer plugin by manually tracing neurites from the Soma to the distal tip. Primary neurite number was quantified in ImageJ by counting neurites directly extending from the Soma. Only processes longer than 10 μm were included in the analysis. For analyses focusing on neuronal subtype identity (Figure 2), marker expression was quantified relative to MAP2^+^ or GABA^+^ neurons. All image acquisition and quantitative analyses were conducted in a blinded manner.

### RNA sequencing and bioinformatics analysis

Total RNA was isolated from AMnp- and AMmnp-induced fibroblast-derived neuronal cultures using TRIzol reagent according to the manufacturer’s instructions. RNA integrity was assessed prior to library preparation. Strand-specific RNA sequencing libraries were constructed using the TruSeq Stranded RNA Library Prep Kit (Illumina) and sequenced on an Illumina NovaSeq 6,000 platform with the SP Reagent Kit to generate 50 bp paired-end reads. All sequencing procedures were performed at the Jinfeng Laboratory Single-Cell Sequencing Center.

Raw sequencing reads were subjected to quality control and adaptor trimming, followed by alignment to the human reference genome (hg38) using HISAT2. Gene-level read counts were quantified with featureCounts. Differential gene expression analysis between AMnp and AMmnp samples was carried out using the DESeq2 package (version 1.50.2). Genes with an absolute log_2_ fold change ≥1 and an adjusted *P* value <0.05 were considered significantly differentially expressed. Differential expression results were visualized using EnhancedVolcano (version 1.28.2) and heatmaps generated with ggplot2 (version 4.0.1) and ComplexHeatmap (version 2.26.0) packages.

To characterize biological processes and pathways associated with transcriptional remodeling, Gene Ontology (GO) enrichment analysis was performed using the ClusterProfiler package (version 4.18.4). Enrichment analyses were conducted separately for upregulated and downregulated gene sets, revealing activation of neuronal differentiation-related programs and repression of extracellular matrix-associated processes in AMmnp cells. In addition, pathway activity inference was performed using PROGENy (version 1.32.0), enabling estimation of signaling pathway activities based on transcriptomic signatures. Distinct pathway activity profiles were observed between AMnp and AMmnp states.

To further explore higher-order transcriptional organization, weighted gene co-expression network analysis (WGCNA, version 1.73) was performed to identify gene modules correlated with neuronal reprogramming states. Module-trait relationships were assessed, and hub genes within representative modules were identified. Functional annotation of selected modules was carried out using GO and KEGG pathway enrichment analyses, highlighting modules associated with extracellular matrix organization, cytoskeletal regulation, and neuronal differentiation. Transcription factor expression dynamics associated with neuronal reprogramming were examined by extracting and visualizing a curated set of transcription factors with established roles in cell fate specification and neuronal differentiation. Expression patterns were displayed as row-scaled heatmaps to emphasize relative differences between AMnp and AMmnp samples.

Alternative splicing (AS) events were systematically analyzed using rMATS and LeafCutter to detect differential exon usage between AMnp and AMmnp conditions. Splicing events were classified into major categories, including skipped exons (SE), alternative 5′splice sites (A5SS), alternative 3′splice sites (A3SS), mutually exclusive exons (MXE), and retained introns (RI). Differential percent spliced-in (dPSI) values were calculated to quantify splicing changes.

### Semiquantitative RT-PCR for splicing analysis

Total RNA was isolated with TRIzol™ reagent (Thermo Fisher Scientific, cat. No. 15596026) in accordance with the manufacturer’s guidelines. Reverse transcription was performed using HiScript® IV All-in-One Ultra RT SuperMix (Vazyme, cat. No. R43301) to generate complementary DNA. GAPDH was used as an internal reference for normalization across samples. Primer sequences designed to distinguish exon inclusion and exclusion isoforms are listed in Supplementary Table S1.

Semiquantitative PCR amplification was carried out using Taq DNA Polymerase (Vazyme, cat. No. P10103). Amplified products were separated on 3% agarose gels and visualized under UV illumination. Band intensities corresponding to exon inclusion and exclusion isoforms were quantified using Quantity One software (Bio-Rad). Percent spliced-in (PSI) values were calculated as the ratio of the inclusion isoform intensity to the sum of inclusion and exclusion isoform intensities. All splicing assays were performed in three independent biological replicates to ensure reproducibility.

### RNA immunoprecipitation and quantitative real-time PCR

RNA immunoprecipitation (RIP) assays were performed using the Magna RIP™ RNA-Binding Protein Immunoprecipitation Kit (Millipore, cat. No. 17–700) according to the manufacturer’s instructions. Cells were lysed in RIP lysis buffer supplemented with RNase inhibitor (Thermo Fisher Scientific, cat. No. N8080119) and a protease inhibitor cocktail (Thermo Fisher Scientific, cat. No. 87785). An aliquot corresponding to 10% of the total lysate was retained as input control and processed in parallel for RNA extraction. The remaining lysates were incubated overnight at 4 °C with magnetic beads conjugated to either an MBNL1-specific antibody (Proteintech, cat. No. 66837-1-Ig, 1:50) or normal mouse IgG (Proteintech, cat. No. B900620, 1:50) as a negative control. Following extensive washing, RNA associated with immunoprecipitated ribonucleoprotein complexes was purified using RNAiso Plus reagent. RNA isolated from immunoprecipitates and input samples was reverse-transcribed using All-in-One Ultra RT SuperMix (Vazyme, cat. No. R43301). Quantitative real-time PCR was carried out using SYBR Green Master Mix (Vazyme, cat. No. Q111-03) on a CFX Opus 96 Real-Time PCR Detection System (Bio-Rad).

To assess MBNL1 binding to *NUMA1* pre-mRNA, primer sets were designed to amplify regions proximal to exon 16, including the upstream intronic region, the intronic sequence immediately downstream of exon 16, and a distal intronic region outside the exon 16 regulatory context. This design enabled evaluation of site-specific MBNL1 association with *NUMA1* transcripts during alternative splicing regulation. Primer sequences are listed in Supplementary Table S1. Ct values obtained from immunoprecipitated RNA were normalized to the corresponding input fractions, with input Ct values adjusted according to the proportion of lysate used (ΔCt = Ct__IP_ − Ct__Input, adjusted_). Enrichment of *NUMA1* transcripts in MBNL1 immunoprecipitates was calculated relative to IgG controls using the comparative Ct method (ΔΔCt = ΔCt__MBNL1_ − ΔCt__IgG_), and results are presented as fold enrichment (2^−ΔΔCT^). All RIP experiments were performed with at least three independent biological replicates.

### Statistical analysis

Sample sizes were determined based on established field standards and prior publications; no formal power analysis was performed before experimentation. Quantitative data are presented as mean ± standard deviation (SD). Data distributions were assessed for normality using the Shapiro-Wilk test. For pairwise comparisons, two-tailed unpaired Student’s *t*-tests were used when data met assumptions of normality and equal variance; otherwise, Welch’s *t*-test was applied. Comparisons involving multiple groups were analyzed using one-way analysis of variance (ANOVA) with Tukey’s *post hoc* test when variances were equal, or Welch’s ANOVA followed by the Games-Howell multiple-comparisons test when variances were unequal. Statistical significance was defined as *P* < 0.05. No formal outlier detection was performed, and all data points were retained for analysis. Statistical analyses were conducted using GraphPad Prism version 8.0.

## Results

### AMmnp induction enhances neurite outgrowth without significantly altering conversion efficiency

**FIGURE 1:**
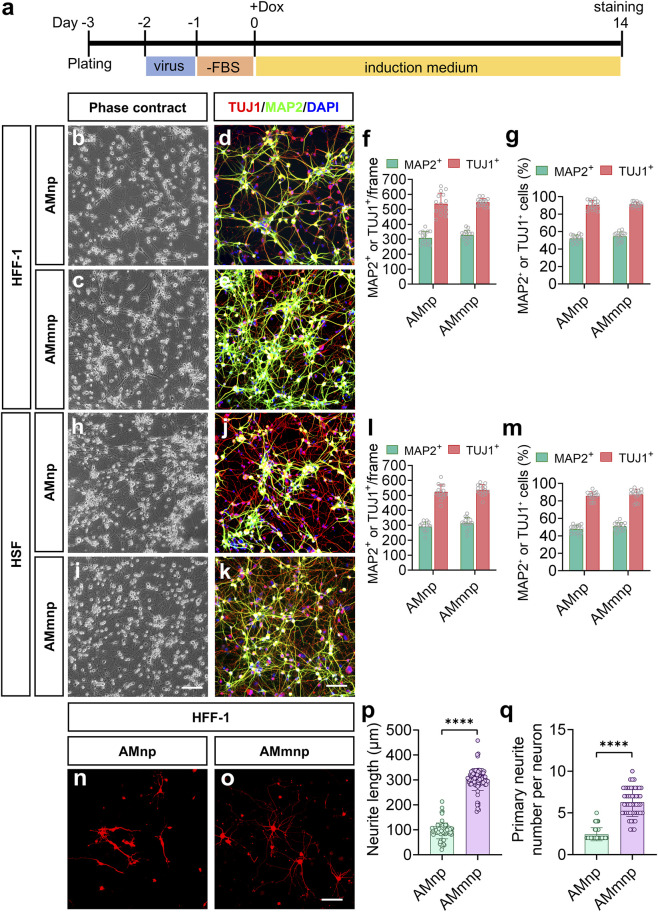
MBNL1 knockdown enhances neuronal outgrowth during fibroblast-to-neuron reprogramming. **(a)** Schematic illustration of the experimental design and treatment timeline for AMnp (ASCL1 + miR-9/9*-124 + *PTBP2* and *p53* knockdown) and AMmnp (AMnp + *MBNL1* knockdown) cells during neuronal induction. **(b,c)** Phase-contrast images of human fibroblasts (HFF-1) at Day 14 after neuronal induction under the indicated conditions. Scale bars, 100 μm. **(d,e)** Representative immunofluorescence images of AMnp and AMmnp cells derived from HFF-1 fibroblasts, stained for the neuronal markers MAP2 and TUJ1, with DAPI counterstaining. **(f,g)** Quantification of the number of MAP2^+^ or TUJ1^+^ cells per field **(f)** and the percentage of MAP2^+^ or TUJ1^+^ cells among DAPI^+^ cells **(g)** in HFF-1-derived neurons. **(h,i)** Phase-contrast images of human fibroblasts (HSF) at Day 14 after neuronal induction under the indicated conditions. Scale bars, 100 μm. **(j,k)** Representative immunofluorescence images of AMnp and AMmnp cells derived from HSF fibroblasts, stained for the neuronal markers MAP2 and TUJ1, with DAPI counterstaining. **(l, m)** Quantification of the number of MAP2^+^ or TUJ1^+^ cells per field **(l)** and the percentage of MAP2^+^ or TUJ1^+^ cells among DAPI^+^ cells **(m)** in HSF-derived neurons. **(n,o)** Representative immunofluorescence images showing MAP2-positive neuronal processes in AMnp and AMmnp cells derived from HFF-1 cells. **(p)** Quantification of neurite length, measured as the longest neurite per neuron. Data were obtained from three independent experiments, with a total of 90 neurons analyzed per condition. **(q)** Quantification of the number of primary neurites per neuron in AMnp- and AMmnp-induced neurons. Only neurites longer than 10 μm were included in the analysis. Data are presented as mean ± SD. Statistical significance was determined using Student’s t-test. ****P < 0.0001.

To establish and compare distinct neuronal induction states, cells were subjected to the AMnp or AMmnp reprogramming regimen according to the timeline shown in Figure 1a. Briefly, cells were plated on day −3, transduced with virus on day −2, cultured under serum-free conditions on day −1, and induced with doxycycline (Dox) beginning on day 0, followed by maintenance in induction medium until endpoint staining on day 14 (Figure 1a). Neuronal induction outcomes were assessed by immunofluorescence staining at Day 14 for the neuronal markers MAP2 and TUJ1, with DAPI counterstaining. In HFF-1 cells, both AMnp and AMmnp induction generated cells exhibiting neuronal-like morphology and expressing MAP2 and TUJ1 (Figures 1b–e). Quantitative analysis revealed that the number of MAP2^+^ or TUJ1^+^ cells per field was comparable between AMnp and AMmnp conditions (Figure 1f). Consistently, the percentage of MAP2^+^ or TUJ1^+^ cells among DAPI^+^ nuclei did not show a significant difference between the two groups (Figure 1g), indicating that AMmnp induction does not substantially alter overall neuronal conversion efficiency relative to AMnp.

To determine whether these observations were reproducible across different fibroblast cell lines, we performed parallel analyses in adult HSF cells (Figures 1h–k). Similar to HFF-1 cells, AMnp and AMmnp induction yielded comparable numbers and percentages of MAP2^+^ and TUJ1^+^ cells (Figures 1l,m). These results indicate that AMmnp does not increase the efficiency of neuronal fate conversion across different cellular contexts. Separation of individual fluorescence channels further confirmed the specificity and distribution of MAP2 and TUJ1 signals under AMnp and AMmnp conditions in both HFF-1 and HSF cells (Supplementary Figure S1). To further assess neuronal maturation, we performed immunostaining for NeuN (RBFOX3), a marker of mature neurons. Compared with AMnp-induced neurons, AMmnp-induced neurons exhibited a significantly higher percentage of NeuN^+^ cells among MAP2^+^ neurons (Supplementary Figure S2), further supporting enhanced neuronal maturation in the AMmnp group.

Notably, despite comparable neuronal conversion efficiency, AMmnp-induced neurons displayed markedly more elaborate neuronal morphology. Representative MAP2-stained images revealed longer neuronal processes in the AMmnp group compared with AMnp (Figures 1n,o). Quantitative measurement of neurite length confirmed a significant increase in the length of the longest neurite per neuron in AMmnp-induced cells (Figure 1p). Beyond the increase in neurite length, AMmnp-induced neurons displayed a more complex multipolar morphology. Quantitative analysis demonstrated a significant increase in the number of primary neurites per neuron, suggesting enhanced neuronal polarization and structural maturation under AMmnp conditions (Figure 1q). Together, these results demonstrate that AMmnp induction enhances neurite outgrowth and neuronal morphological complexity without substantially affecting the overall efficiency of neuronal conversion.

### AMmnp induction results in a GABAergic-enriched neuronal identity

**FIGURE 2:**
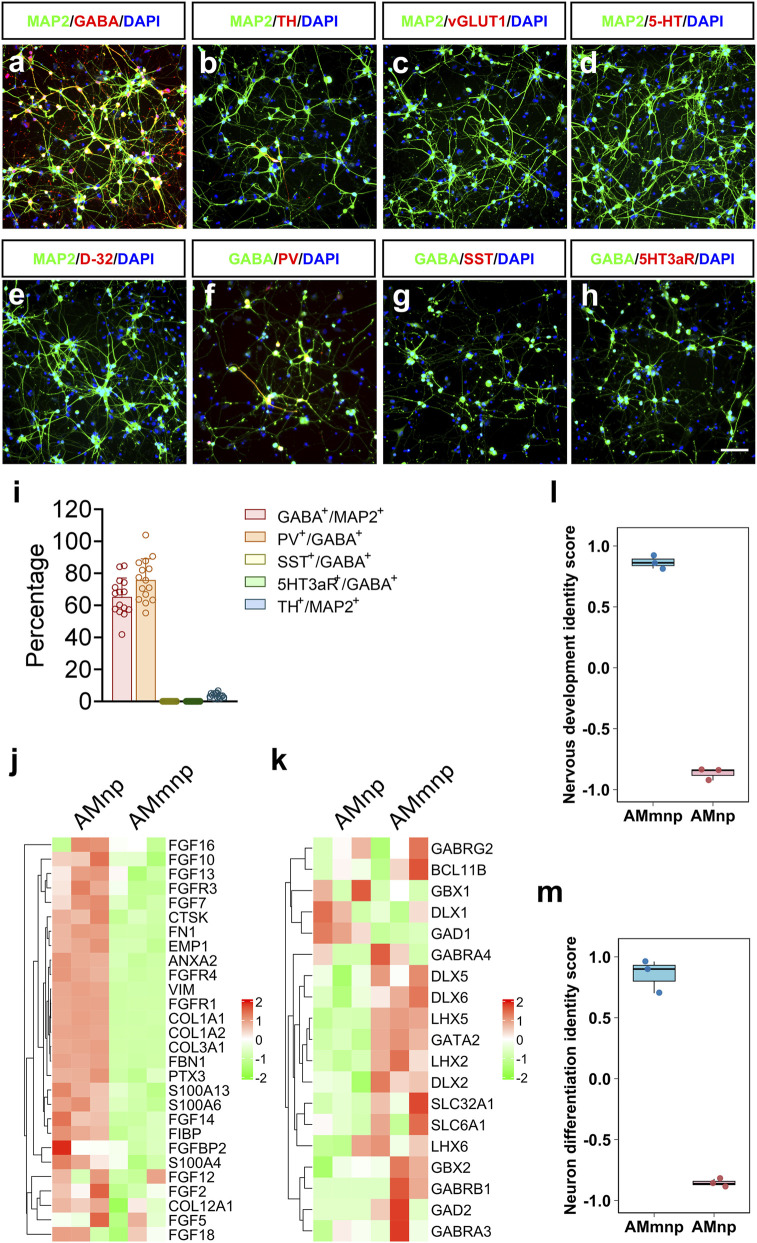
Neuronal subtype specification and transcriptomic identity differences in AMmnp cells. **(a–e)** Representative immunofluorescence images of AMmnp-induced neurons stained for MAP2 in combination with markers of major neuronal subtypes, including GABA **(a)**, TH **(b)**, vGLUT1 **(c)**, 5-HT **(d)**, and DARPP-32 (D-32) **(e)** with DAPI counterstaining. **(f–h)** Representative immunofluorescence images of AMmnp-induced neurons stained for GABA in combination with markers of inhibitory neuronal subtypes, including PV **(f)**, SST **(g)**, 5HT3aR **(h)** with DAPI counterstaining. Scale bars, 100 μm. **(i)** Quantification of neuronal subtype composition, expressed as the percentage of indicated subtype marker-positive cells among MAP2^+^ or GABA^+^ neurons, as specified. **(j,k)** Heatmaps showing the relative expression of selected gene sets associated with fibroblast identity **(j)** and inhibitory neuron-related identity markers **(k)** across AMnp and AMmnp samples. **(l, m)** Box plot summarizing the nervous system development and neuronal differentiation identity score in AMnp and AMmnp samples. Each dot represents an individual biological replicate. Data are presented as mean ± SD.

To define the neuronal subtype identities associated with the AMmnp state, we first performed immunofluorescence-based characterization of neuronal subtype composition. These included GABA (inhibitory neurons), tyrosine hydroxylase (TH; dopaminergic), vesicular glutamate transporter 1 (vGLUT1; glutamatergic), serotonin (5-HT; serotonergic), and DARPP-32 (D-32; medium spiny-like neurons) (Figures 2a–e; Supplementary Figure S3). Notably, GABA-positive neurons constituted the predominant population, whereas TH^+^, vGLUT1^+^, 5-HT^+^, and DARPP-32^+^ neurons were detected only sporadically (Figures 2a–e). Further analysis of inhibitory neuronal diversity revealed that GABA^+^ neurons co-expressing parvalbumin (PV), somatostatin (SST), or 5-HT3aR were readily observed, indicating the emergence of inhibitory neuron populations expressing interneuron-associated markers following AMmnp induction (Figures 2f–h; Supplementary Figure S3). Quantitative assessment confirmed the predominance of inhibitory neuronal identity: GABA^+^ neurons accounted for approximately 65.3% of the MAP2^+^ neuronal population, whereas TH^+^/MAP2^+^ neurons represented only 3.8% (Figure 2i). Within the GABAergic population, PV^+^ neurons comprised 75.9% of GABA^+^ cells. Together, these findings demonstrate that AMmnp induction results in a neuronal identity enriched for GABAergic features rather than a uniform enhancement of differentiation across multiple neuronal subtypes.

### Transcriptomic signatures reveal enhanced neuronal differentiation and inhibitory programs in AMmnp cells

To further investigate the molecular basis underlying the observed phenotypic differences, we analyzed transcriptomic profiles of AMnp and AMmnp samples. Heatmap analysis of selected fibroblast-related gene sets revealed a concerted downregulation of fibroblast identity programs in AMmnp relative to AMnp cells (Figure 2j). Consistent with the immunofluorescence results, genes associated with inhibitory neuron function and GABAergic signaling were enriched in AMmnp cells, providing transcriptomic support for the GABAergic phenotype observed by immunostaining (Figure 2k). At the pathway and biological process level, gene expression-based scoring further supported these observations. AMmnp exhibited significantly higher nervous development identity scores compared with AMnp (Figure 2l). Likewise, neuron differentiation identity scores were markedly increased in AMmnp-induced cells (Figure 2m), indicating a transcriptional shift toward a more neuron-like differentiated state rather than an increase in neuronal conversion efficiency.

Together, these results indicate that AMmnp induction is associated with a GABAergic-enriched transcriptional and phenotypic profile and coordinated activation of transcriptional programs linked to neuronal differentiation.

### Global transcriptomic remodeling distinguishes AMmnp from AMnp states

**FIGURE 3:**
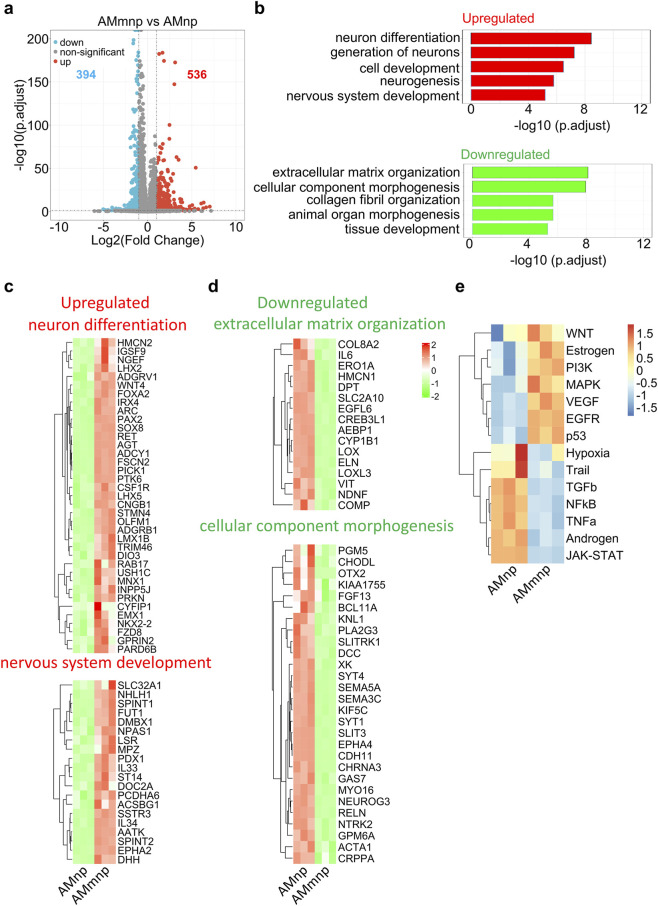
Transcriptomic differences and pathway signatures associated with AMnp and AMmnp states. **(a)** Volcano plot showing differentially expressed genes (DEGs) between AMmnp and AMnp samples identified by RNA sequencing. Genes significantly upregulated in AMmnp cells (log_2_ fold change ≥1, adjusted *P* < 0.05) are shown in red, downregulated genes (log_2_ fold change ≤ −1, adjusted *P* < 0.05) are shown in blue, and non-significant genes are shown in grey. **(b)** Gene Ontology (GO) enrichment analysis of DEGs, highlighting biological processes associated with genes upregulated (top, red) or downregulated (bottom, green) in AMmnp cells relative to AMnp cells. **(c)** Heatmap showing the relative expression of representative genes upregulated in AMmnp cells and enriched in neuronal differentiation and nervous system development-related categories. **(d)** Heatmap showing the relative expression of representative genes downregulated in AMmnp cells and enriched in extracellular matrix organization and cellular component morphogenesis-related categories. **(e)** Heatmap showing PROGENy-inferred signaling pathway activity scores across AMnp and AMmnp samples, with values scaled by row.

To characterize transcriptional differences between AMnp- and AMmnp-induced cells, we performed RNA sequencing and compared global gene expression profiles between the two conditions. Differential expression analysis identified widespread transcriptional changes in AMmnp relative to AMnp, including 536 upregulated and 394 downregulated genes (Figure 3a; Supplementary Table S2). Gene Ontology (GO) enrichment analysis of genes upregulated in AMmnp cells revealed strong enrichment for neuronal differentiation-related biological processes, including neuron differentiation, neurogenesis, and nervous system development (Figure 3b; Supplementary Table S2). Consistent with these enrichments, heatmap visualization demonstrated coordinated upregulation of representative neuronal differentiation genes, together with concordant expression changes in multiple transcription factors implicated in neuronal differentiation and cell fate regulation (Figure 3c; Supplementary Figure S4). In contrast, genes downregulated in AMmnp cells were significantly enriched for GO terms related to extracellular matrix organization, collagen fibril organization, cellular component morphogenesis, and tissue development (Figure 3b). Heatmap analysis further confirmed a concerted reduction in extracellular matrix-associated genes and morphogenetic regulators in AMmnp (Figure 3d), consistent with repression of mesenchymal and structural gene programs associated with fibroblast identity.

At the pathway and network levels, PROGENy-based inference revealed distinct signaling activity profiles between AMnp and AMmnp samples, with multiple pathways exhibiting consistent differences between the two states (Figure 3e). Weighted gene co-expression network analysis (WGCNA) identified gene modules negatively associated with the AMmnp state (Supplementary Figure S5a) that were enriched for extracellular matrix organization and cytoskeletal regulation (Supplementary Figures S5b–d) and showed coordinated downregulation, consistent with differential expression analysis. Notably, hub gene analysis of the ME1 (turquoise) module highlighted coordinated downregulation of genes involved in cytoskeletal organization, extracellular matrix remodeling, metabolic homeostasis, and signal transduction (Supplementary Table S3).

Together, these network-level changes indicate that the AMmnp state is characterized by systematic repression of fibroblast-associated structural and extracellular matrix programs, thereby creating a transcriptional landscape more permissive for neuronal differentiation.

### AMmnp state is associated with widespread alternative splicing remodeling across neuron-related genes

**FIGURE 4:**
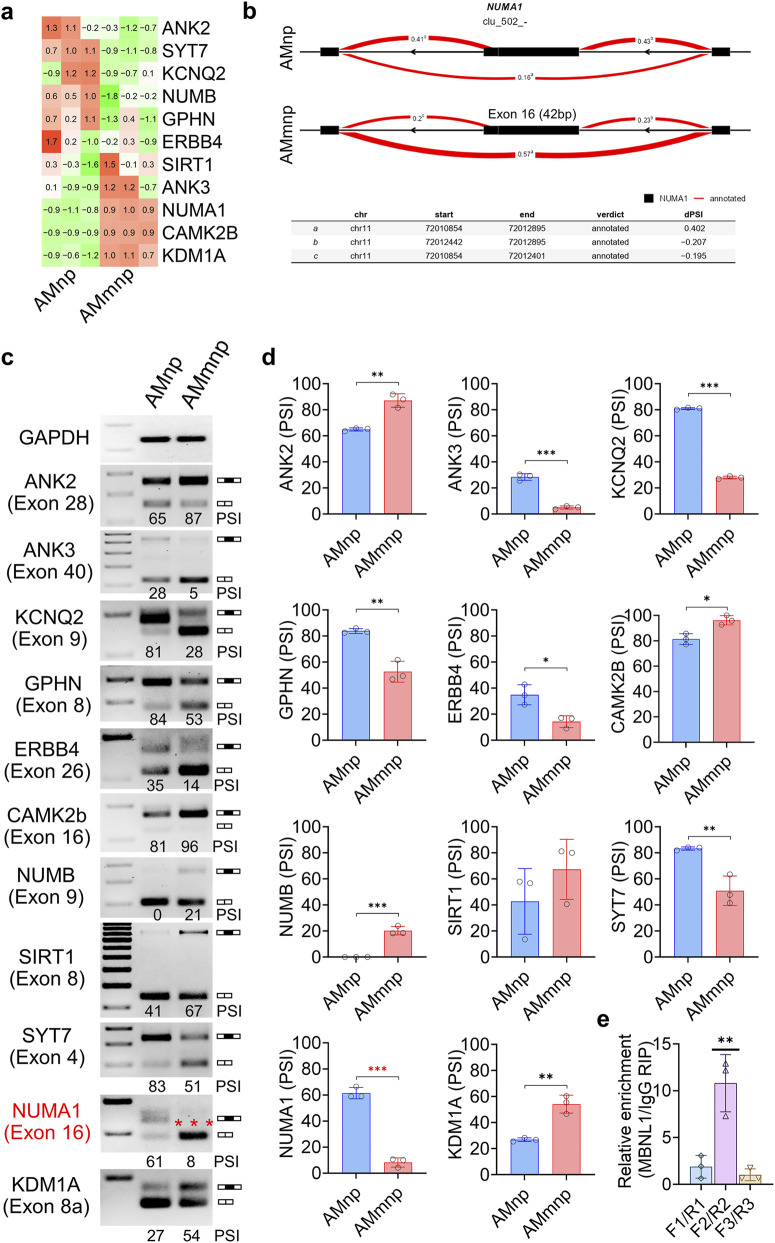
Alternative splicing remodeling associated with distinct AMnp and AMmnp cellular states. **(a)** Heatmap showing row-scaled z-scores of PSI values for significantly altered alternative splicing events between AMnp and AMmnp samples. **(b)** Schematic representation of *NUMA1* alternative splicing patterns illustrating differential inclusion or skipping of exon 16 (42 bp) between AMnp and AMmnp conditions. Exons are shown as boxes and introns as connecting lines. Red arcs indicate splice junction usage, with arc thickness reflecting relative junction abundance. Differential splicing is quantified by changes in percent spliced-in (ΔPSI) values. **(c)** RT-PCR validation of representative alternative splicing events identified by transcriptomic analysis. Representative gel images are shown for selected genes, including *ANK2, ANK3, KCNQ2, GPHN, ERBB4, CAMK2B, NUMB, SIRT1, SYT7, NUMA1,* and *KDM1A*, with inclusion and exclusion isoforms indicated. Corresponding PSI values are shown below each lane. **(d)** Quantification of PSI values for the alternative splicing events shown in panel **(c)**. Bars represent mean PSI ±SD derived from independent experiments. Statistical significance between AMnp and AMmnp samples is indicated. **(e)** RNA immunoprecipitation (RIP) analysis assessing the association between MBNL1 and *NUMA1* exon 16-proximal pre-mRNA. Relative enrichment was quantified by qPCR and normalized to IgG controls, supporting a direct interaction between *MBNL1* and *NUMA1* transcripts at the exon 16 region. Data are presented as mean ± SD. Statistical significance was determined using Student’s t-test. *P < 0.05, **P < 0.01, ***P < 0.001.

Given the extensive transcriptional reprogramming observed in AMmnp cells (Figure 3), we next examined whether alternative splicing contributes to the establishment of distinct neuronal states. Comparative splicing analysis revealed widespread differences in exon usage between AMnp and AMmnp samples, affecting numerous genes implicated in neuronal excitability, synaptic organization, and signal transduction (Figure 4a). Global classification of alternative splicing events revealed that exon skipping (SE) was the predominant splicing alteration (n = 2,376), followed by mutually exclusive exons (MXE, n = 459), alternative 3′splice sites (A3SS, n = 430), alternative 5′splice sites (A5SS, n = 390), and retained introns (RI, n = 282) (Supplementary Figure S6). Notably, many differentially spliced genes (DSGs) encode proteins involved in neuronal function, including *ANK2, ANK3, KCNQ2, GPHN, ERBB4, CAMK2B, NUMB, SIRT1, SYT7, NUMA1,* and *KDM1A* (Figure 4a).

Differential splicing analysis using the LeafCutter framework identified multiple high-confidence splicing events between AMnp and AMmnp conditions. Among these, *NUMA1* exhibited a prominent and condition-specific switch in exon 16 usage, with exon inclusion favored in AMnp cells and pronounced exon skipping in AMmnp cells (Figure 4b). This event was characterized by opposing ΔPSI values across alternative exon-skipping junctions, consistent with strong regulation of *NUMA1* exon 16 splicing during AMmnp induction.

To validate the transcriptome-derived splicing changes, we performed RT-PCR analysis on a panel of representative targets. Consistent with RNA-seq results, AMmnp cells displayed coordinated alterations in exon inclusion across multiple neuronal genes, including reduced exon inclusion in *ANK3, KCNQ2, GPHN, ERBB4, SYT7,* and *NUMA1*, and increased inclusion in *ANK2, CAMK2B, NUMB, SIRT1,* and *KDM1A* (Figures 4c,d). Quantification of percent spliced-in (PSI) values confirmed statistically significant differences in exon usage between AMnp and AMmnp states for all examined targets. Furthermore, RNA immunoprecipitation assays revealed significant enrichment of *NUMA1* exon 16-proximal transcripts in MBNL1 immunoprecipitates compared with IgG controls (Figure 4e), indicating direct interaction between MBNL1 and *NUMA1* pre-mRNA.

Together, these results demonstrate that the AMmnp state is characterized by extensive alternative splicing remodeling across neuron-related genes and identify the MBNL1-NUMA1 axis as a key regulator of neuronal identity during reprogramming. Consistent with these findings, functional enrichment analysis of DSGs revealed significant enrichment of processes related to actin filament organization, supramolecular fiber organization, and cell projection dynamics, along with RNA splicing pathways (Supplementary Figure S7), supporting a role for alternative splicing in coordinating structural remodeling and neuronal phenotypic output during reprogramming.

### *NUMA1* exon 16 alternative splicing differentially modulates neuronal phenotypic output during AMmnp-induced reprogramming

**FIGURE 5:**
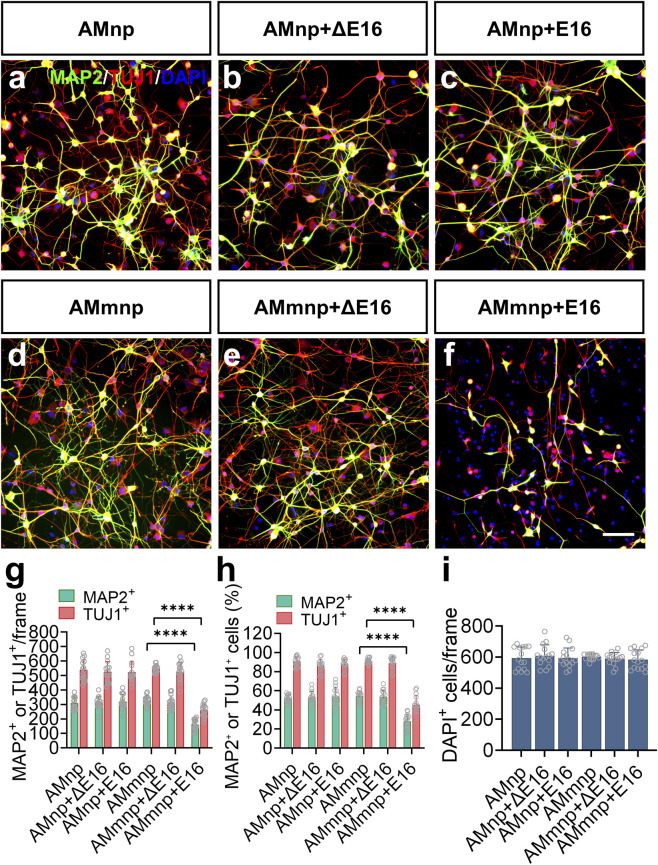
*NUMA1* exon 16 alternative splicing modulates neuronal phenotypic output during reprogramming. **(a–f)** HFF-1 cells were subjected to neuronal reprogramming under the indicated conditions for 14 days. Representative immunofluorescence images show cells expressing AMnp or AMmnp alone, or together with *NUMA1* splice isoforms containing exon 16 (E16) or lacking exon 16 (ΔE16). Cells were co-stained for the neuronal markers MAP2 and TUJ1, with DAPI counterstaining, to assess neuronal marker expression and morphological features associated with neuronal differentiation. **(g)** Quantification of the number of MAP2^+^ or TUJ1^+^ cells per field corresponding to panels **(a–f)**. **(h)** Quantification of the percentage of MAP2^+^ or TUJ1^+^ cells among DAPI^+^ nuclei corresponding to panels **(a–f)**. **(i)** Quantification of the total number of DAPI^+^ cells per field corresponding to panels **(a–f)**, indicating comparable overall cell numbers across experimental conditions. Data are presented as mean ± SD from multiple independent experiments. Statistical significance was determined using one-way ANOVA followed by *post hoc* multiple-comparison tests. Scale bars, 100 μm ****P < 0.0001.

To assess the functional impact of *NUMA1* exon 16 alternative splicing on neuronal reprogramming outcomes, we manipulated *NUMA1* splice isoform expression during AMnp- and AMmnp-mediated neuronal induction. HFF-1 cells were reprogrammed under AMnp or AMmnp conditions in the presence of *NUMA1* constructs either lacking exon 16 (ΔE16) or containing exon 16 (E16), followed by immunofluorescence analysis of MAP2 and TUJ1 expression (Figures 5a–f; Supplementary Figure S8). Under the AMnp condition, expression of either the ΔE16 or exon 16-containing *NUMA1* isoform did not significantly alter MAP2^+^ or TUJ1^+^ cells compared with AMnp alone (Figures 5a–c,g,h). In contrast, in the AMmnp context, expression of the ΔE16 isoform did not further enhance neuronal marker levels, whereas reintroduction of the exon 16-containing isoform resulted in a significant reduction in MAP2^+^ and TUJ1^+^ neuronal cells (Figures 5d–h), consistent with a context-dependent effect of *NUMA1* exon 16 inclusion on neuronal marker acquisition. The total number of DAPI^+^ cells remained comparable across all experimental conditions (Figure 5i), indicating that the observed differences in MAP2^+^ and TUJ1^+^ neuronal populations were unlikely to result from major alterations in overall cell survival or proliferation.

**FIGURE 6:**
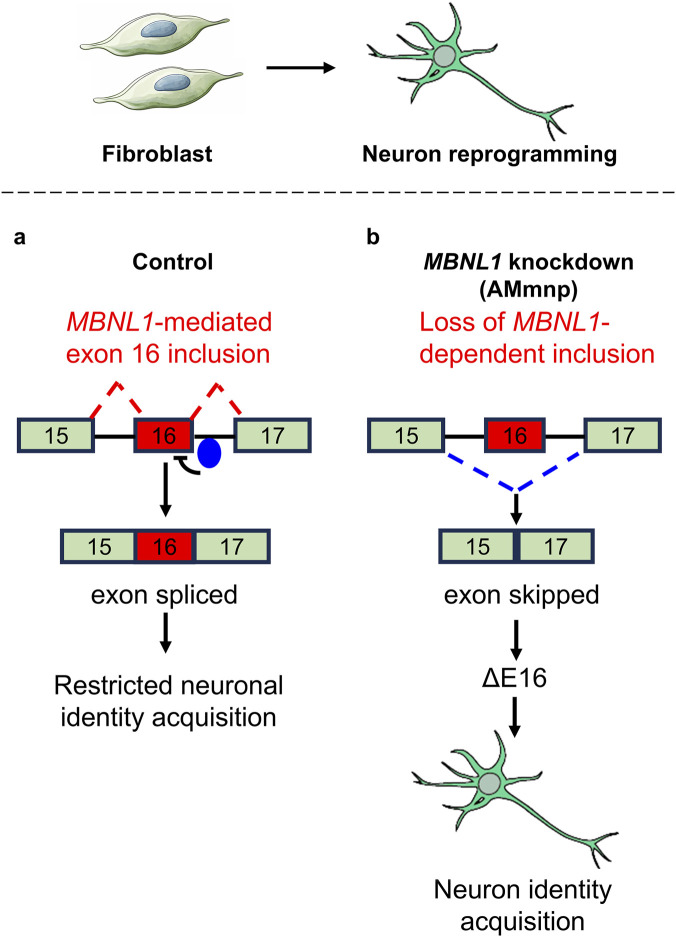
Model illustrating *MBNL1*-dependent regulation of *NUMA1* exon 16 alternative splicing during neuronal reprogramming. **(a)** In control cells, *MBNL1* promotes inclusion of *NUMA1* exon 16, resulting in exon-included transcripts. This splicing pattern is associated with a more restricted neuronal identity acquisition during reprogramming. **(b)** Upon *MBNL1* knockdown (AMmnp state), *MBNL1*-dependent exon 16 inclusion is reduced, leading to exon skipping and generation of the ΔE16 *NUMA1* isoform. This splicing switch is associated with a neuronal state more permissive for neuronal identity acquisition and maturation. Exons are depicted as boxes and introns as connecting lines. Solid lines indicate exon inclusion, whereas dashed lines indicate exon skipping.

Quantitative analyses based on both absolute numbers and proportions of marker-positive cells confirmed these effects, while total DAPI^+^ cell counts remained comparable across all conditions, excluding major contributions from altered cell survival or proliferation. Together, these data show that *NUMA1* exon 16 alternative splicing selectively modulates neuronal phenotypic output downstream of the AMmnp state. Exon 16 skipping is associated with enhanced neuronal marker expression, whereas exon inclusion exerts an opposing suppressive effect. Consistent with these observations, MBNL1-dependent regulation of *NUMA1* exon 16 splicing emerges as a key splicing event linked to neuronal identity acquisition during reprogramming (Figure 6).

## Discussion

Direct fibroblast-to-neuron reprogramming provides a powerful system to interrogate mechanisms governing neuronal fate acquisition beyond transcription factor-driven lineage conversion (Wang et al., 2021; Bocchi et al., 2022). In this study, we show that knockdown of the RNA-binding protein *MBNL1* establishes a distinct reprogramming state (AMmnp) characterized by a GABAergic-enriched neuronal identity, enhanced neurite outgrowth, and extensive remodeling of gene regulatory programs. However, *MBNL1* depletion did not significantly alter neuronal conversion efficiency. Consistent with previous single-cell transcriptomic analyses showing that later reprogramming states can diverge substantially without changes in initial fate induction or conversion efficiency (Treutlein et al., 2016), AMmnp represents a distinct transcriptional state associated with altered neuronal phenotypic properties. Together, our findings indicate that MBNL1 does not control whether fibroblasts become neurons, but instead governs how neuronal identity is refined following fate induction through coordinated transcriptional and splicing regulation.

Transcriptomic analyses revealed that AMmnp cells undergo coordinated repression of extracellular matrix, cytoskeletal, and mesenchymal identity programs, accompanied by activation of neuronal differentiation pathways. WGCNA further identified gene modules strongly associated with the AMmnp state that were enriched for extracellular matrix organization, focal adhesion, and developmental processes, with hub genes such as *VIM, CALD1, COL1A1, COL3A1,* and *LTBP2* showing concerted downregulation (Treutlein et al., 2016; Sonsalla et al., 2024). These results suggest that dismantling structural and mechanical programs that stabilize fibroblast identity may create a permissive cellular environment for neuronal phenotypic elaboration during reprogramming (Wang et al., 2021; Bocchi et al., 2022). Notably, AMmnp-induced neurons also displayed enhanced multipolar morphology and increased primary neurite formation. Since neuronal polarization is an early feature of neuronal maturation, these morphological changes further support a more advanced differentiation state induced by AMmnp.

Beyond transcriptional remodeling, extensive alternative splicing changes emerged as a defining feature of the AMmnp state, with exon skipping representing the dominant splicing outcome following *MBNL1* knockdown. Among the affected targets, *NUMA1* exon 16 stood out as a prominent and functionally relevant MBNL1-associated target. Previous large-scale splicing analyses identified *NUMA1* as a direct splicing target of *MBNL1*, in which loss of *MBNL1* promotes exon 16 skipping (Sebestyen et al., 2016). Our results extend this regulatory relationship to a neuronal reprogramming context and support a functional contribution of MBNL1-dependent *NUMA1* exon 16 splicing to neuronal identity acquisition.

Functional experiments revealed that manipulation of *NUMA1* exon 16 does not significantly affect neuronal marker expression under AMnp conditions but exerts pronounced effects specifically in the AMmnp background. This context-dependent effect suggests that *NUMA1* exon 16 skipping is not sufficient on its own to drive neuronal reprogramming but becomes functionally relevant once the MBNL1-dependent transcriptional and splicing landscape has been established. While expression of the ΔE16 isoform failed to further enhance neuronal marker output once the AMmnp state was established, reintroduction of the exon 16-containing isoform significantly reduced neuronal marker expression. Although the downstream mechanism remains to be fully defined, NUMA1 is well positioned to influence neuronal phenotypic elaboration through its structural roles in microtubule organization, spindle-associated architecture, and cytoskeletal coupling (Kiyomitsu and Boerner, 2021; Cho et al., 2025). In the AMmnp background, exon 16 inclusion may preserve a structural state less permissive for neurite elaboration and neuronal morphological maturation. This interpretation is also consistent with the broader repression of extracellular matrix and cytoskeletal programs observed in AMmnp cells. We note, however, that the current isoform-rescue strategy represents a gain-of-function approach and does not fully recapitulate endogenous regulation of *NUMA1* exon 16 splicing. Therefore, although these data support an isoform-dependent effect in the AMmnp context, future studies using endogenous splice-switching approaches, such as antisense oligonucleotides (ASO) or CRISPR-based splice modulation, will be needed to define the physiological contribution of *NUMA1* exon 16 more precisely.

An important limitation of the present study is that neuronal maturation was primarily evaluated based on morphological features, neuronal marker expression, and transcriptomic signatures, whereas functional electrophysiological characterization was not performed. Therefore, although AMmnp-induced cells exhibited enhanced neurite complexity and increased expression of neuronal differentiation-associated markers, the extent to which these cells acquire fully functional neuronal properties remains to be determined. Future studies incorporating patch-clamp electrophysiology and synaptic activity analyses will be important for defining the functional maturation state of AMmnp-induced neurons more comprehensively.

Rather than altering neuronal conversion efficiency, our study uncovers a post-transcriptional regulatory layer that governs neuronal identity refinement after fate induction. We identify MBNL1 as a central node linking repression of fibroblast-associated structural programs to alternative splicing control of cytoskeletal regulators, with the MBNL1-NUMA1 exon 16 axis providing evidence for a mechanistic link between splicing decisions and neuronal phenotypic output. Consistent with this model, functional enrichment analysis of DSGs further indicates enrichment of processes related to cytoskeletal organization and cell projection dynamics, along with RNA processing pathways, supporting a coordinated role for alternative splicing in shaping structural and phenotypic aspects of neuronal differentiation. Together, these findings position alternative splicing as an active determinant of neuronal identity, linking MBNL1-dependent splicing control to structural remodeling and phenotypic refinement during reprogramming.

## Data Availability

The data presented in the study are deposited in the Gene Expression Omnibus (GEO) repository, accession number GSE317293.

## Glossary

AMnp: ASCL1 + miR-9/9*-124 + *PTBP2* and *p53* knockdown
AMmnp: ASCL1 + miR-9/9*-124 + *PTBP2* + *p53* and *MBNL1* knockdown
ANK2: Ankyrin 2
ANK3: Ankyrin 3
CAMK2B: Calcium/calmodulin-dependent protein kinase II beta
DARPP-32: Dopamine- and cAMP-regulated phosphoprotein of 32 kDa
DEG: Differentially expressed genes
DSG: Differentially spliced genes
ECM: Extracellular matrix
ERBB4: Erb-B2 receptor tyrosine kinase 4
GABA: γ-Aminobutyric acid
GEO: Gene Expression Omnibus
GO: Gene Ontology
GPHN: Gephyrin
HSF: Human skin fibroblasts
HFF-1: Human foreskin fibroblast-1
KCNQ2: Potassium voltage-gated channel subfamily Q member 2
KDM1A: Lysine demethylase 1 A
MAP2: Microtubule-associated protein 2
MBNL1: Muscleblind-like splicing regulator one
NUMA1: Nuclear mitotic apparatus protein one
PV: Parvalbumin
PSI: Percent spliced-in
RIP: RNA immunoprecipitation
RNA-seq: RNA sequencing
SIRT1: Sirtuin one
SST: Somatostatin
SYT7: Synaptotagmin seven
TH: Tyrosine hydroxylase
TUJ1: Neuron-specific class III β-tubulin
vGLUT1: Vesicular glutamate transporter one
5-HT: 5-hydroxytryptamine
5HT3aR: 5-hydroxytryptamine receptor 3 A
