# Supplementary material for: MBNL1-dependent alternative splicing promotes neuronal differentiation through regulation of NUMA1 exon 16 during fibroblast-to-neuron reprogramming
Source: Front Cell Dev Biol. 2026 Jun 22;14:1862147. doi: 10.3389/fcell.2026.1862147 (PMC13333663; doi:10.3389/fcell.2026.1862147)
Supplement: Supplementary file 4 [file DataSheet1.docx]

##### **Supplementary Information for**

**MBNL1-dependent alternative splicing promotes neuronal differentiation through regulation of NUMA1 exon 16 during fibroblast-to-neuron reprogramming**

Jun Li^1^, Qiu-Shuang Long^2^, Ruo-Qi Zhang^2^, Bing-Lin Zhu^2,^ *

^1^ Brain Research Center and State Key Laboratory of Trauma, Burns, and Combined Injury, the Army Medical University (Third Military Medical University), Chongqing, 400038, China

^2^ Jinfeng Laboratory, Chongqing, 401329, China

* Correspondence and requests for materials should be addressed to:
Bing-Lin Zhu, Ph.D.,
Jinfeng Laboratory, Chongqing, 401329, China
Email: zhubinglin@jflab.ac.cn

**Contents**

**Figure S1. Separate fluorescence channels corresponding to Figure 1.**

**Figure S2. AMmnp-induced neurons exhibit increased NeuN expression.**

**Figure S3. Separate fluorescence channels corresponding to Figure 2.**

**Figure S4. Transcription factor expression dynamics associated with the AMmnp state.**

**Figure S5. Co-expression network analysis identifies gene modules associated with the AMmnp state.**

**Figure S6. Global landscape of alternative splicing changes upon *MBNL1* knockdown.**

**Figure S7. Functional enrichment analysis of differentially spliced genes (DSGs).**

**Figure S8. Separate fluorescence channels corresponding to Figure 5.**

**Table S1. Primer sequences used for PCR or RT-qPCR.**

**Table S2. Differentially expressed genes (DEGs) in induced neurons.**

**Table S3. Hub genes of the ME1 (turquoise) module identified by WGCNA analysis.**

**Figure S1**


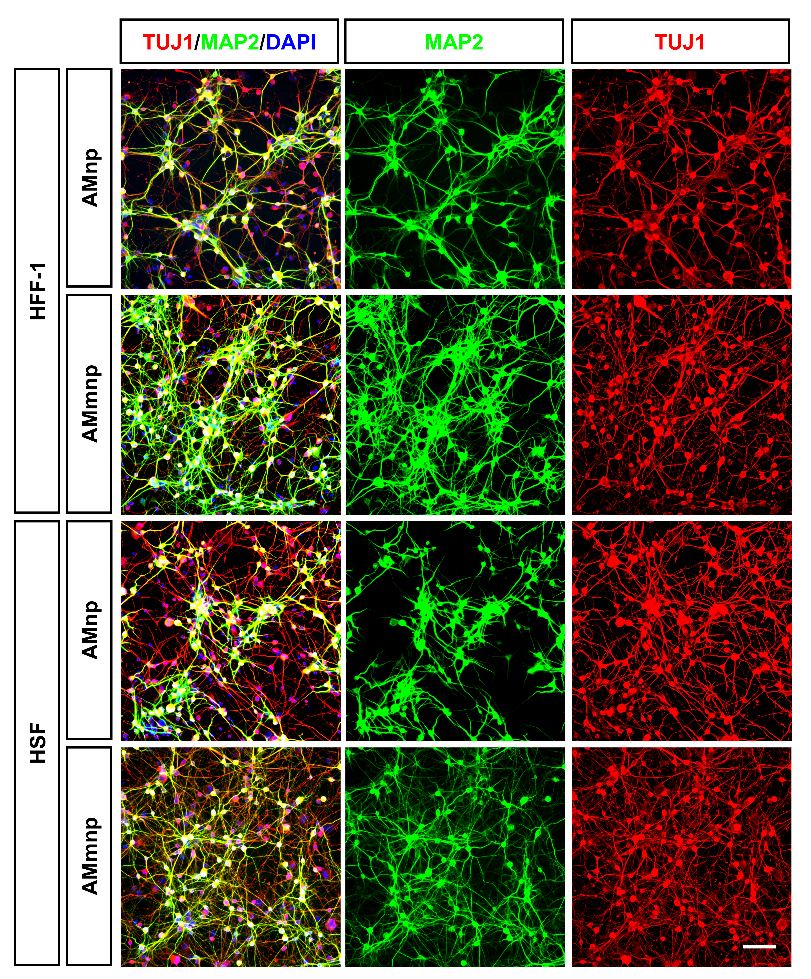


Representative immunofluorescence images showing separated fluorescence channels for TUJ1 (red), MAP2 (green), and DAPI (blue) corresponding to Figure 1. Images are shown for AMnp and AMmnp conditions under HFF-1 and HSF fibroblast backgrounds, as indicated. These single-channel images highlight differences in neuronal marker expression and neurite architecture between conditions. Scale bars, 100 μm.

**Figure S2**


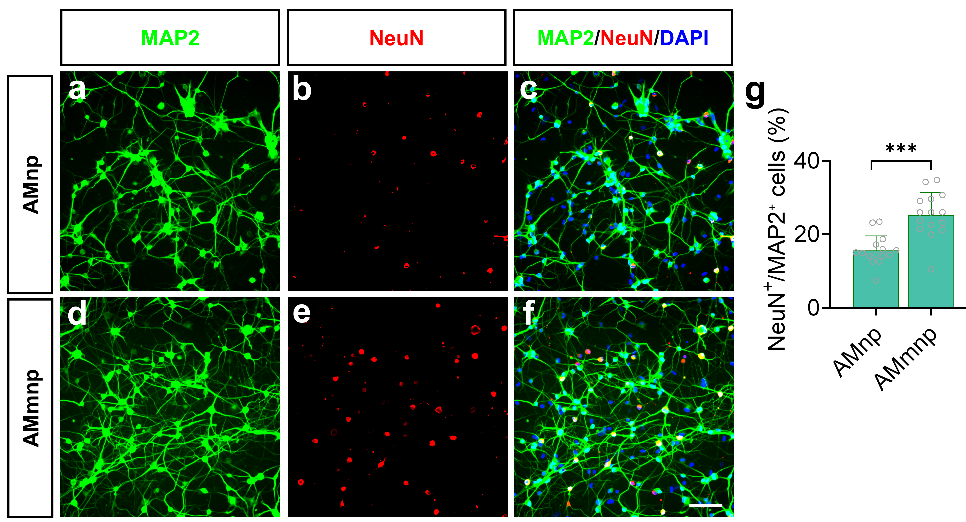


(a-f) Representative immunofluorescence images of MAP2 (green), NeuN (red), and DAPI (blue) staining in AMnp- and AMmnp-induced neurons at day 14 after neuronal induction.

(g) Quantification of the percentage of NeuN-positive cells among MAP2-positive neurons in HFF-1-derived neurons.

Data are presented as mean ± SD from three independent experiments. ***P < 0.001 by Student’s t-test. Scale bar, 100 μm.

**Figure S3**


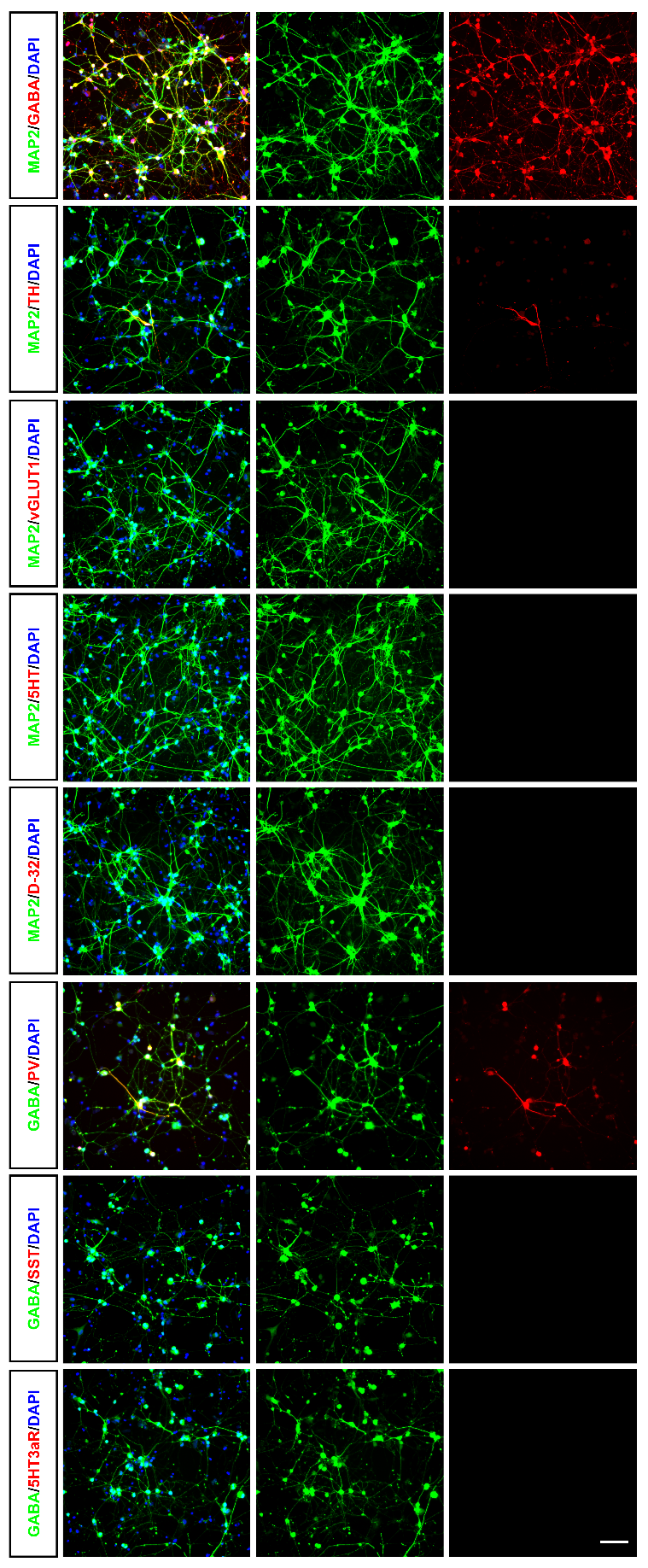


Representative immunofluorescence images showing separated fluorescence channels corresponding to the merged images in Figure 2. Individual channels for MAP2, neuronal subtype markers, GABA, TH, vGLUT1, 5-HT, DARPP-32 (D-32), PV, SST, 5HT3aR, and DAPI are shown as indicated. These single-channel images illustrate marker specificity, signal distribution, and neuronal subtype-associated expression patterns under the indicated conditions. Scale bars, 100 μm.

**Figure S4**


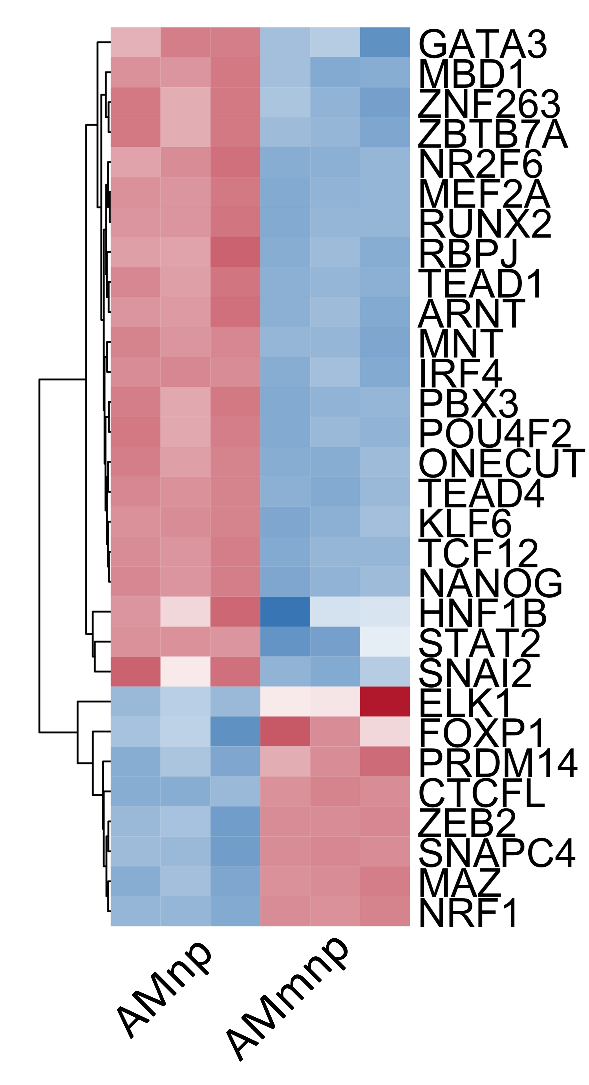


Heatmap showing the expression patterns of selected transcription factors across AMnp and AMmnp samples. Transcription factors were selected based on differential expression analysis and reported involvement in cell fate regulation, transcriptional control, or neuronal lineage-associated programs. Expression values are scaled by row to emphasize relative differences between conditions. Hierarchical clustering was applied to genes to reveal shared expression trends across biological replicates.

**Figure S5**


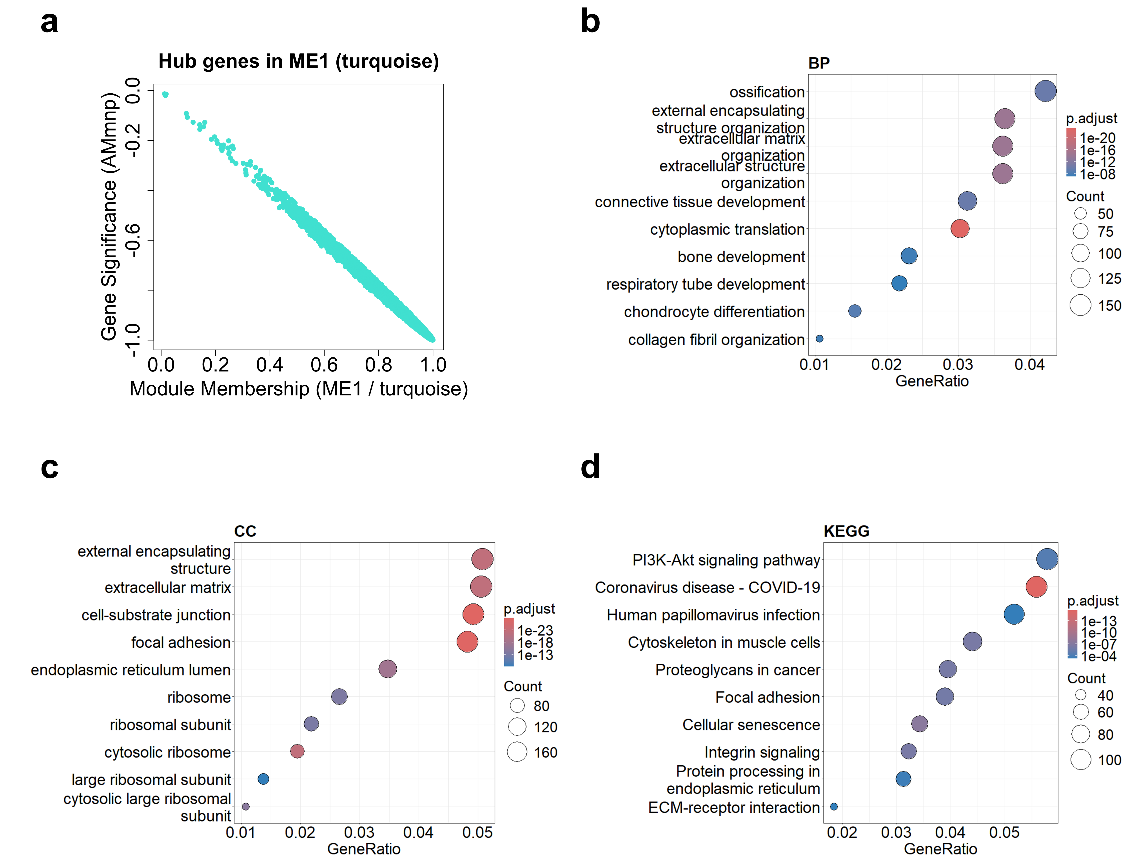


(a) Scatter plot showing the relationship between module membership (MM) and gene significance (GS) for genes in the ME1 (turquoise) module identified by weighted gene co-expression network analysis (WGCNA). Each dot represents an individual gene.

(b) Gene Ontology (GO) biological process (BP) enrichment analysis of genes within the ME1 (turquoise) module, visualized as a dot plot. The x-axis indicates the gene ratio, dot size represents the number of genes, and color denotes adjusted *P* values.

(c) Gene Ontology (GO) cellular component (CC) enrichment analysis of the ME1 (turquoise) module, displayed as a dot plot using the same visualization scheme as in (b).

(d) KEGG pathway enrichment analysis of genes in the ME1 (turquoise) module, highlighting pathways related to extracellular matrix organization, focal adhesion, and related signaling processes.

**Figure S6**


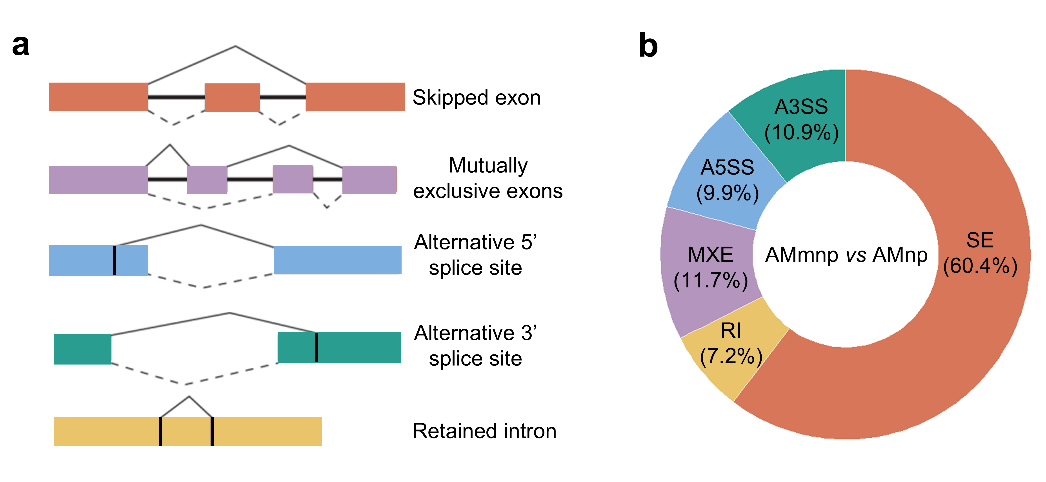


(a) Schematic overview illustrating the major categories of alternative splicing events detected upon *MBNL1* knockdown, including skipped exons (SE), alternative 3′ splice sites (A3SS), alternative 5′ splice sites (A5SS), mutually exclusive exons (MXE), and retained introns (RI). Solid lines indicate canonical splicing patterns, whereas dashed lines indicate alternative splicing events.

(b) Pie chart summarizing the relative distribution of alternative splicing event types identified between AMmnp and AMnp samples following *MBNL1* knockdown. Event counts are as follows: skipped exons (SE, n = 2376), alternative 5′ splice sites (A5SS, n = 390), alternative 3′ splice sites (A3SS, n = 430), mutually exclusive exons (MXE, n = 459), and retained introns (RI, n = 282).

**Figure S7**


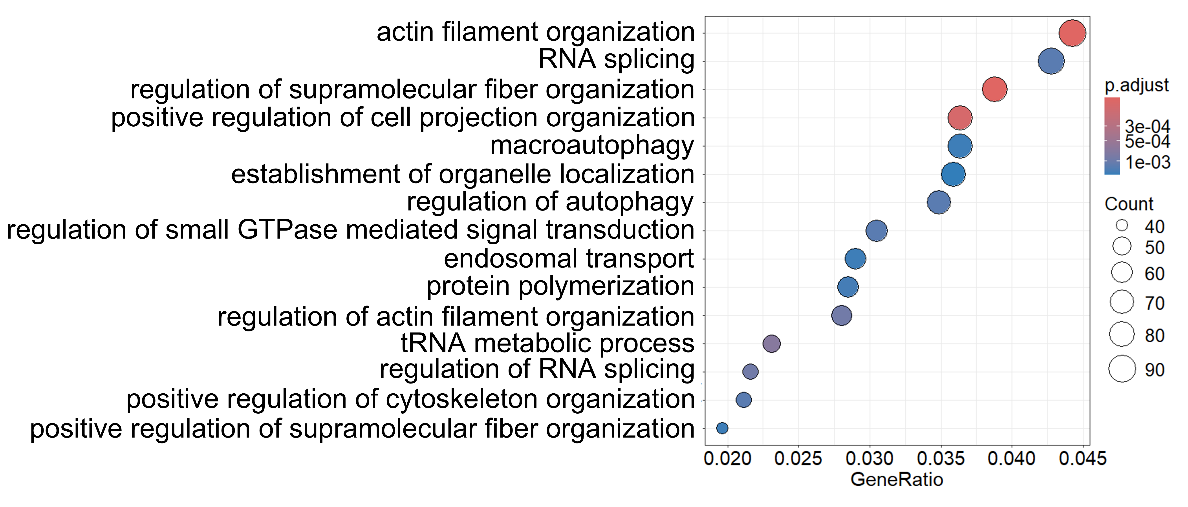


Gene Ontology (GO) biological process enrichment analysis was performed on differentially spliced genes (DSGs) identified between AMnp and AMmnp conditions. The dot plot shows significantly enriched GO terms ranked by gene ratio. Dot size indicates the number of genes associated with each term, and color represents adjusted *P* values. Enriched terms highlight biological processes related to actin filament dynamics, cell projection organization, and RNA splicing regulation.

**Figure S8**


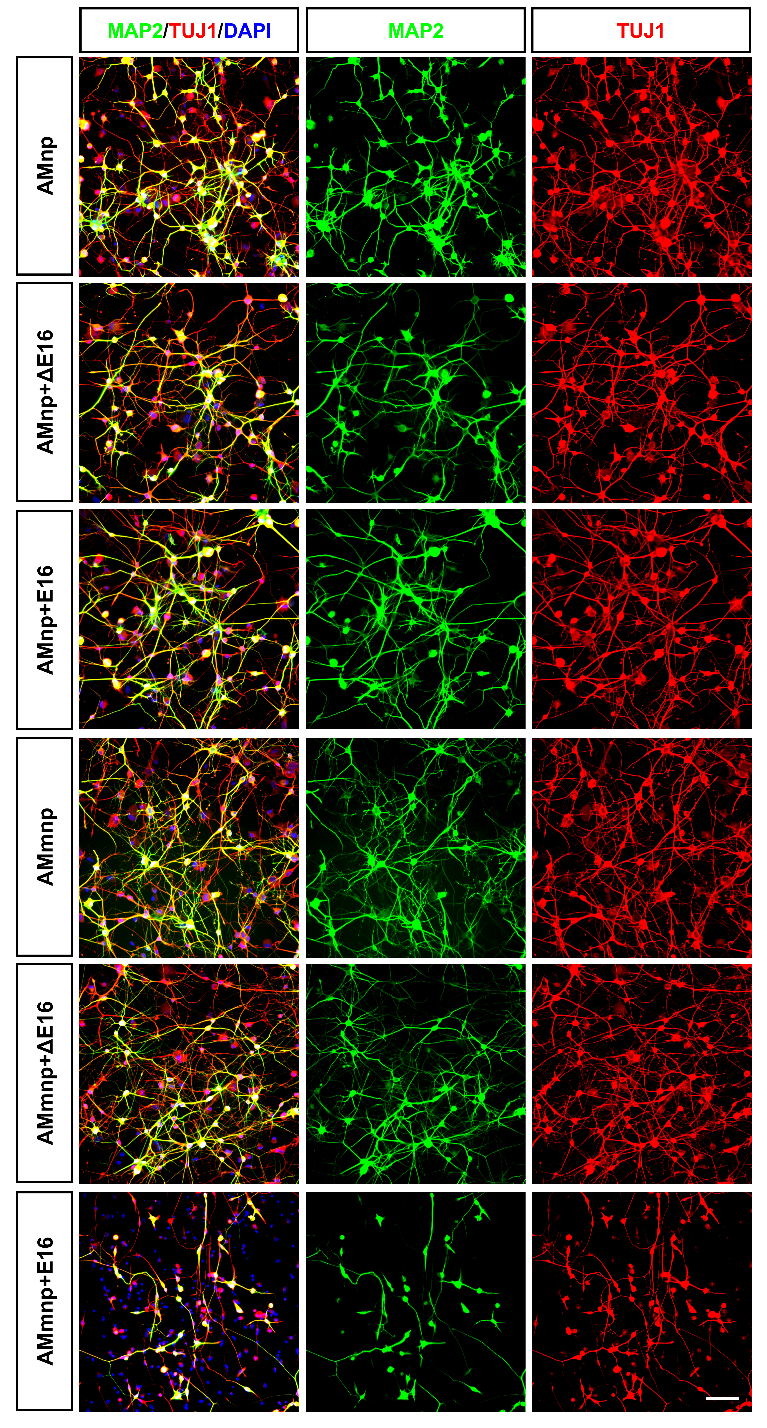

Representative immunofluorescence images showing separated fluorescence channels corresponding to the merged images in Figure 5. Images display individual channel signals for MAP2 (green), TUJ1 (red), and DAPI (blue). HFF-1 cells were reprogrammed under AMnp or AMmnp conditions, with or without expression of *NUMA1* splice isoforms containing exon 16 (E16) or lacking exon 16 (ΔE16). These single-channel images illustrate neuronal marker distribution, neurite morphology, and relative neuronal marker abundance under the indicated conditions. Scale bars, 100 μm.
